# Quality-by-Design approach to the fluid-bed coating of ginkgo lactone nanosuspensions

**DOI:** 10.1039/c8ra03288b

**Published:** 2018-06-15

**Authors:** Jiawei Han, Xin Wang, Jingxian Wang, Lingchong Wang, Lihua Chen, Junsong Li, Wen Li

**Affiliations:** College of Pharmacy, Nanjing University of Chinese Medicine 138 Xianlin Avenue Nanjing 210023 PR China lijunsong1964@163.com +86-25-85811517 +86-25-85811517; Jiangsu Provincial TCM Engineering Technology Research Center of High Efficient Drug Delivery System (DDS) Nanjing 210023 PR China; College of Pharmacy, Jiangxi University of Traditional Chinese Medicine 18 Yunwan Road Nanchang 330004 PR China

## Abstract

The Quality-by-Design (QbD) approach was employed to investigate the fluid-bed coating process for the conversion of ginkgo lactone (GL) liquid nanosuspensions into dried nanosuspensions. The effects of critical process variables including inlet air temperature, inlet air capacity and atomizing air pressure were investigated. The particle size and percent yield were optimized using a full factorial design. A Box-Behnken design (BBD) was employed to generate the response surface and optimize process conditions. Multi-linear regression and one-way ANOVA were used to analyze the relationship between critical variables and responses. The results showed that all three selected variables were significant factors (*p* < 0.05) affecting the particle size. Higher inlet temperature, inlet air capacity or atomizing air pressure will cause an increase of particle size. In addition, the percent yield primarily depended on the inlet air temperature and inlet air capacity (*p* < 0.05). A higher percent yield was obtained at a higher inlet air temperature or inlet air capacity. The optimal conditions for BBD, including inlet air temperature, inlet air capacity and atomizing air pressure, were set at 40 °C, 11.6 Nm^3^ and 0.7 bar, respectively. Compared with the raw GLs, the optimized products presented an amorphous state and possessed much faster dissolution. The particle size, percent yield, PDI, zeta-potential and redispersibility index of the optimized products were 254.3 ± 9.8 nm, 82.36 ± 1.87%, 0.155 ± 0.02, −32.9 ± 3.8 mV and 113 ± 4.4% (*n* = 3), respectively. These results indicate that fluid-bed coating technology based on a QbD approach was sufficient for the solidification of nanosuspensions.

## Abstract 2

The Quality-by-Design (QbD) approach was employed to investigate the fluid-bed coating process for the conversion of ginkgo lactone (GL) liquid nanosuspensions into dried nanosuspensions.

## Graphical abstract

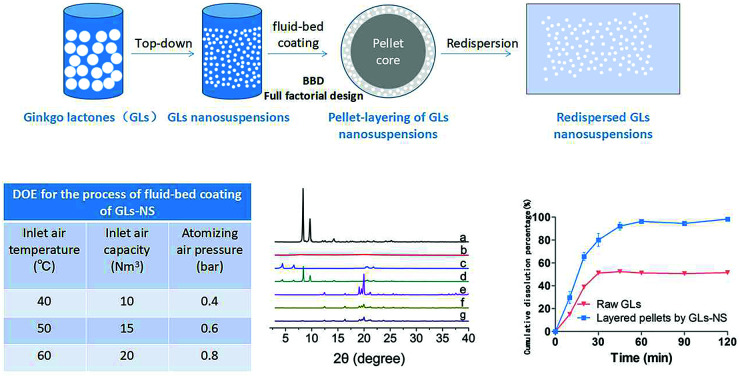

## Introduction

Nanosuspensions are colloidal dispersions of nanosized drug particles stabilized by surfactants. These dispersions can also be defined as biphasic systems comprising pure drug particles dispersed in aqueous media.^1^ A significant drawback of nanosuspensions is the physical and chemical instability of these materials in aqueous media, including agglomeration, sedimentation and crystalline transformation.^2^ Therefore, the solidification of nanosuspensions is considered as a key step in the production of the final nanosuspension form intended for oral delivery.

Solidification methods for nanosuspensions typically include freeze-drying, spray drying, vacuum drying, fluid-bed drying, *etc.*^3–6^ Among these methods, fluid-bed coating is a “one-step” technique commonly used to add a film coating onto a substrate, and has widespread applications in the pharmaceutical industry. Fluid-bed coating involves the evaporation of a solvent and the simultaneous deposition of coating materials onto the surface of nonpareil pellets. Compared with conventional spray drying, fluid-bed coating is applicable at relatively lower temperatures and is less costly and time-consuming. Moreover, fluid-bed coating is more scalable.^7,8^ Thus, the solidification of nanosuspensions by fluid-bed coating is attractive and promising. However, the solidification of drug nanosuspensions by fluid-bed coating is also challenging because solidified formulations need to have the ability to reconstitute into their original nanosuspensions. Any material undergoing a drying process experiences significant stress. For example, a reduction in the solvent volume can lead to a decrease in the solubility of the surfactant or stabilizer, resulting in precipitation and rendering these unavailable for protection of the nanoparticles against aggregation. There are only a few studies^7,9–11^ on the fluid-bed drying of nanosuspensions, but no exhaustive study has been conducted to understand this process, particularly the impact of technological factors on redispersibility and percent yield.

Quality-by-Design (QbD) is a versatile and systematic approach mentioned in various documents of the International Conference on Harmonization (ICH) guidelines.^12–14^ This approach emphasizes the design/development and manufacture of formulations to ensure predefined product quality objectives.^15^ One of the important components of QbD is Design of Experiment (DoE).^16^ DoE is used to confirm significant and non-significant factors affecting product quality attributes. Moreover, this technique can be used to evaluate the relationships between those factors and define a product quality design space.^17^

Ginkgo lactones (GLs) have recently attracted considerable attention.^18^ These natural lactones could bind to the membrane receptors, inhibit the platelet-activating factor, and produce anticoagulant effect. GLs have long been used to protect against neural damage in a variety of circumstances.^19^ However, the poor water-solubility and low oral bioavailability of these natural compounds have greatly limited the formulation development and clinical application of GLs. Previous studies have demonstrated that nanosuspensions could enhance the oral bioavailability and bio-efficacy of GLs.^20^

Hence, the aim of the present study was to convert liquid nanosuspensions into dried nanosuspensions using fluid-bed coating technology. DoE, including a full factorial design and Box–Behnken, was employed in the present study. A full factorial design was used to understand the effects of the critical process parameters of fluid-bed coating and select the main factors with significant effects. Box–Behnken design (BBD) is a response surface methodology combining mathematical and statistical methods. BBD is used to establish the model and analyze multiple variables, and the objective is to optimize process conditions. Inlet air temperature, inlet air capacity and atomizing air pressure were selected as critical process variables, whereas particle size and percent yield were examined as responses. ANOVA and multifactorial analysis were performed to elucidate the interactions between critical variables, to rank order the critical variables, and to provide a predictive model for pellet-coating using fluid-bed technology. The particle size, redispersibility index (RDI), differential scanning calorimetry (DSC), power X-ray diffraction (PXRD), scanning electron microscopy (SEM) and *in vitro* dissolution were employed to characterize and evaluate dried nanosuspensions under optimal conditions.

## Materials and methods

### Materials

**Fig. 1:**
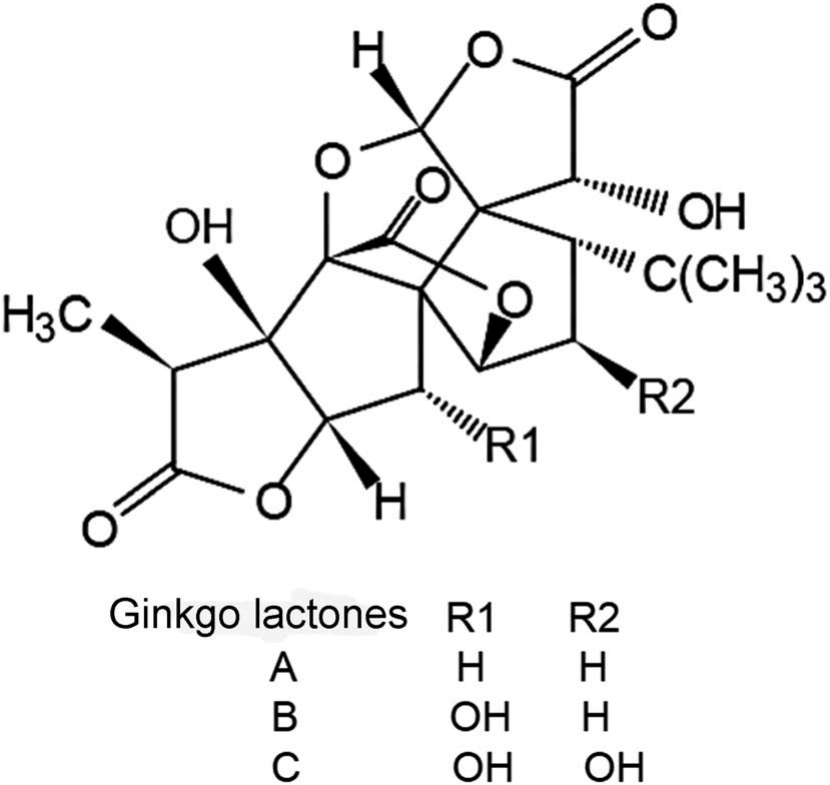
Chemical structures of ginkgo lactones.

GLs containing ginkgolide A (GA) (35.0%) and ginkgolide B (GB) (62.2%) were purchased from Nanjing Zixi Biological Products Co., Ltd (Nanjing, China), and chemical structures of ginkgo lactones are shown in Fig. 1. Hydroxypropyl methyl cellulose (HPMC) was provided by BASF (Ludwigshafen, Germany). Sodium dodecyl sulfate (SDS) was provided by Aladdin Industrial Co., Ltd. (Shanghai, China). Microcrystalline cellulose (MCC) pellets (0.50–0.71 mm in diameter) were purchased from Gaocheng Biotech and Health Co, Ltd (Hangzhou, China). Deionized water was prepared using the Synergy® UV water purification system (Millipore, Billerica, MA, USA). Other reagents were of analytical grade.

### Preparation of liquid ginkgo lactones nanosuspensions

The liquid GLs nanosuspensions (GLs-NS) were prepared using a wet media milling (top–down) approach. Briefly, 800 mg of raw GLs was dispersed in 200 mL aqueous solution containing 0.2% (w/v) HPMC and 0.2% (w/v) SDS under magnetic stirring. The obtained mixture was disintegrated into microparticles using a high shear homogenizer (BRT, B25, Germany) at 16 000 rpm for 5 min. The resulting suspensions were subsequently wet-milled with zirconium oxide beads (0.3–0.4 mm in diameter) using an N T-0.3 L Mill (Dongguan Longly Machinery Factory, China) and milled for 2 h at 2400 rpm to finally obtain liquid GLs-NS. The processing temperature was maintained at less than 20 °C by passing cooled water through the outer jacket.

### Coating the liquid nanosuspensions onto pellets

The layered pellets were produced by coating the liquid GLs-NS onto MCC pellets as nonpareil pellets using a fluid-bed coater with a bottom spray (Mini-Glatt, Glatt GmbH, Binzen, Germany). Briefly, 2% (w/v) lactose, as a drying protector, was added to liquid GLs-NS. The dispersion was sprayed through a nozzle (0.5 mm in diameter) onto the surface of the MCC pellets in the fluid-bed coater. The fluid-bed coating was pre-conditioned at the required setting of inlet air temperature, inlet air capacity (namely the drying capacity of the fluidized air) and atomizing air pressure. The rotational speed of the peristaltic pump was maintained at 3 rpm. After fluid-bed coating, the pellets were dried in a coating chamber at 30 °C for an additional 15 minutes to obtain the layered pellets. The dried samples were removed from the coating chamber using a plastic scraper and stored between 2 and 8 °C till further analysis.

### Experimental design

Based on preliminary experiments, the inlet air temperature, inlet air capacity and atomizing air pressure were selected as important fluid-bed coating process variables. Besides, polydispersity index (PDI) and zeta-potential of GLs-NS were all less than 0.2 and −30 mV pre- and post-coating, respectively. So particle size and percent yield were selected as evaluation index (data not shown). All liquid GLs-NS were prepared using a wet media milling approach as mentioned above. A full factorial design 3^3^ (3 factors at 3 levels) was used to select significant factors. The three independent variables used at three levels in this investigation were: inlet air temperature (40–60 °C); inlet air capacity (10–20 Nm^3^); and atomizing air pressure (0.4–0.8 bar). Four center points were added to the design space to identify non-linearity relationship in the responses. All operating conditions were confirmed as achievable. All experiments were completely randomized to reduce systematic errors, and 200 mL of liquid GLs-NS were used for coating under different processing conditions (according to design space). Multi-linear regression and ANOVA were performed to analyze the relationships between critical variables and responses.

Based on a full factorial design, a response surface methodology was adopted to optimize the coating process using the BBD. The whole design comprised 17 experimental points, and the experiment was conducted in random order. All trials were performed in triplicate, and each trial was optimized using critical response parameters, such as *Y*_1_: particle size; *Y*_2_: percent yield.

All experimental design and data analysis were achieved using Design-Expert 8 (Stat-ease®) software.

### Particle size analysis of reconstituted nanosuspensions

The layered pellets (1 g) were dispersed in 20 mL of purified water, and the mixture was stirred with a paddle at 100 rpm for 5 min. Prior to measurement, the samples were passed through a 50-mesh sieve to remove the MCC inert cores, since MCC pellet did not dissolve and disintegrate in contact with purified water.^21,22^ Preliminary experiments revealed that MCC pellet did not affect the reconstitution test.

The particle size and polydispersity index (PDI) were measured using photon correlation spectroscopy (PCS) using a Zetasizer (Nano-ZS90, Malvern Instruments Ltd., Worcestershire, UK) at 25 °C pre- and post-coating. Each sample was analyzed in triplicate, and the results were reported as the mean value of these runs.

### Determination of percent yield

To calculate the percent yield, the drug amount in layered pellets and liquid GLs-NS were determined using an HPLC-UV method (as described for *in vitro* dissolution). The percent yield was calculated using the following formula:

1 Percent yield = (*W*_0_/*W*_1_) × 100

where *W*_0_ and *W*_1_ represents the amount of GLs in layered pellets and liquid GLs-NS, respectively.

### Characterization and *in vitro* evaluation of the dried GLs-NS or layed pellets under optimum conditions

#### Redispersibility study

The redispersibility index (RDI) was expressed as the ratio of the particle size of nanosuspensions pre- and post-fluid-bed coating and calculated using the following formula:

2 RDI = (*D*/*D*_0_) × 100%

where *D*_0_ represents the mean particle size of liquid nanosuspension prior to fluid-bed coating, and *D* is the mean particle size of the nanosuspensions of the redispersible pellets layered by the nanosuspensions. When the RDI value was close to 100%, the pellets were considered completely reconstituted to the original particle size.^23^

#### Scanning electron microscopy

The surface morphology of MCC pellets, the layered pellets and cross-section of MCC pellets and layered pellets was investigated using a SEM (JSM-5610LV, Rigaku, Japan). The samples were glued and mounted onto metal sample plates and subsequently gold-coated using a sputter coater with an electrical potential of 2.0 kV at 30 mA for 240 s. The surface morphology of the layered pellets was examined by operating the SEM at 10 kV.

#### Differential scanning calorimetry

The dried GLs-NS powder (outer layer of layered pellet powder) samples carefully peeled from the outer layer of the zirconium oxide beads were used for physical characterization using DSC and subsequent PXRD analysis. Briefly, the liquid GLs-NS was layered onto zirconium oxide beads (0.3–0.4 mm) using a fluid-bed coater under optimal conditions. The layered zirconium oxide beads were placed in a porcelain mortar and subsequently gently ground to peel off the coating layer. For the control, the blank pellet powder was generated using the same method as employed for the pellet powder without drug. Approximately 6 mg of the samples (raw GLs, HPMC, SDS, physical mixture of GLs with stabilizer, blank pellet powder and dried GLs-NS powder) was weighed into a nonhermetically sealed aluminum pan, and the DSC analysis was performed using a NETZSCH DSC-204 (Netzsch, Selb, Germany). The samples were heated from 30 °C to 350 °C at a heating rate of 10 K per minute. The instrument was calibrated using indium. All DSC measurements were performed in a nitrogen atmosphere at a flow rate of 100 mL per minute.

#### Power X-ray diffraction

The PXRD analysis of the samples (raw GLs, HPMC, SDS, physical mixture of GLs with stabilizer/lactose, blank pellet powder and dried GLs-NS powder) was performed using a diffractometer (D/Max-2500PC, Rigaku, Japan) with a Cu source of radiation. The measurements were obtained at 40 kV and 25 mA. The scanned angle was set from 2° ≤ 2*θ* ≤ 40°, and the scanning rate was 2° min^−1^. The measurements were performed in triplicate.

#### *In vitro* dissolution

The dissolution studies were conducted using Dissolution Test Apparatus III at China Pharmacopoeia.^24^ A ZRS-8G dissolution apparatus (Tianjin Tianda Tianfa Technology Co. Ltd., Tianjin, China) operating at a rotation speed of 50 rpm was used to investigate the *in vitro* dissolution of the raw GLs and layered pellets at 37 ± 0.5 °C, using 250 mL of 0.1 M HCl as the dissolution medium. Samples equivalent to 10 mg GLs were directly added to 250 mL of dissolution medium. All dissolution experiments were performed in triplicate. At each predetermined sampling time, 2 mL of sample was withdrawn using a sampling port fitted with a 0.1 μm filter disc and 2 mL of blank dissolution medium was added back into the vessels through the sampling port. The filtrate was diluted with an equivalent mobile phase. Subsequently, 20 μL was injected into the HPLC for analysis.

HPLC was performed using a Waters 2695 HPLC system (Waters, Milford, UK), equipped with an evaporative light-scattering detector (ELSD). The analytes were separated on a Kromasil C_18_ column (250 × 4.6 mm, 5 μm) maintained at 35 °C. The mobile phase was composed of methanol and water at a ratio of 36/64 (v/v) using an isocratic elution (1.0 mL min^−1^) for 18 min.

## Results and discussion

### Influence of fluid-bed coating variables on aggregation/particle size

As shown in Table 1, the particle size of GLs-NS redispersed from layered pellets varied from 248.10 to 425.30 nm using a full factorial design (PDI less than 0.2). The particle sizes of the liquid GLs-NS prior to coating were approximately 225 nm (PDI less than 0.2).

**Table 1:** Full factorial design space and responses for the process of fluid-bed coating of GLs-NS

Sample number	Experimental conditions			Results	
	Inlet air temperature (°C)	Inlet air capacity (Nm^3^)	Atomizing air pressure (bar)	*Y*_1_: particle size (nm)	*Y*_2_: percent yield
1	40	20	0.4	281.83	78.45
2	50	15	0.6	282.93	78.63
3	60	20	0.8	425.30	65.94
4	60	20	0.4	395.63	67.18
5	60	10	0.4	280.07	78.76
6	60	10	0.8	323.50	70.87
7	40	20	0.8	259.80	79.02
8	40	10	0.4	248.10	83.42
9	40	10	0.8	297.53	78.59
10	50	15	0.6	292.10	75.48
11	50	15	0.6	288.47	74.30
12	50	15	0.6	271.40	79.51

As shown in Table 2, all selected variables, such as the inlet air temperature, inlet air capacity, and atomizing air pressure significantly influenced the particle size (*p* < 0.05).

**Table 2:** Estimated effect of fluid-bed coating variables on particle size

Source	Sum of squares	df	Mean square	*F* value	*p*-value prob > *F*	
Model	28 202.48	5	5640.50	30.58	0.0009	Significant
*A* – inlet air temperature	14 215.80	1	14 215.79	77.06	0.0003	
*B* – inter air capacity	5690.67	1	5690.68	30.85	0.0026	
*C* – atomizing air pressure	1262.53	1	1262.53	6.84	0.0473	
*AB*	6125.40	1	6125.40	33.20	0.0022	
*BC*	908.09	1	908.09	4.92	0.0773	
Curvature	2439.49	1	2439.49	13.22	0.0150	Significant
Residual	922.38	5	184.48			
Lack of fit	677.22	2	338.61	4.14	0.1370	Not significant
Pure error	245.16	3	81.72			
Cor total	31 564.35	11				

**Fig. 2:**
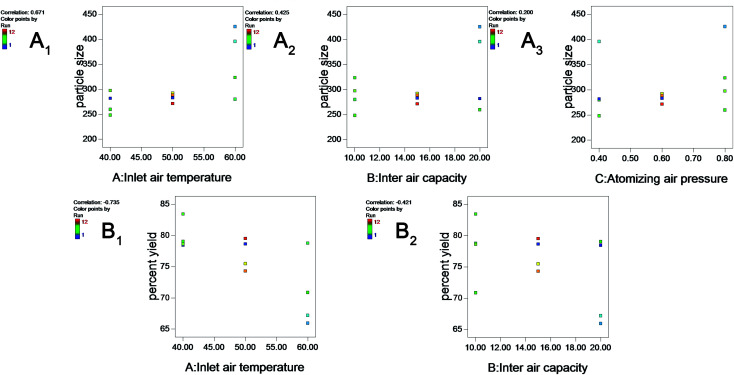
Plots showing the effect of inlet air temperature (A), inlet air capacity (B) and atomizing air pressure (C) on particle size (A_1_–A_3_) and percent yield (B_1_ and B_2_).

Higher inlet temperature induced further aggregation, as shown by the increase in particle size (correlation: 0.671) (Fig. 2A_1_), which might reflect the Ostwald ripening during the evaporation of water.

Fig. 2A_2_ shows that a larger inlet air capacity could increase the size particle (correlation: 0.425). One explanation for this phenomenon might be that the higher inlet air capacity increases the product temperature. In addition, two-way interactions between critical variables in the coating process (inlet air temperature × in inlet air capacity) were significant (*p* < 0.05), reflecting the inter dependency of the variables.

Moreover, Table 2 shows that the curvature is significant (*p* < 0.05), indicating that the effects of these variables might be non-linearity. Therefore, a Box–Behnken design was used to optimize these processes and devices.

### Influence of fluid-bed coating process variables on percent yield

As shown in Table 1, the percent yield varied from 65.94% to 83.42%. Two main factors were significant, inlet air temperature and inlet air capacity (*p* < 0.05), as shown in Table 3. The increase in inlet air temperature and inlet air capacity decreased the percent yield of the layered pellets (the correlations are −0.735 and −0.421, respectively) (Fig. 2B_1_ and B_2_). The observed decrease in yield with increasing inlet air temperature might reflect the fact that a high temperature could accelerate the water evaporation of nanosuspensions, resulting in powder formation and the failure to coat the surface of the pellets. However, an inlet air temperature less than 40 °C could cause the pellets to adhere to each other and decrease the percent yield.

**Table 3:** Estimated effect of fluid-bed coating variables on percent yield

Source	Sum of squares	df	Mean square	*F* value	*p*-value prob > *F*	
Model	0.025	3	0.008	9.96	0.006	Significant
*A* – inlet air temperature	0.017	1	0.017	20.44	0.003	
*B* – inter air capacity	0.006	1	0.006	6.72	0.04	
*C* – atomizing air pressure	0.002	1	0.002	2.72	0.143	
Curvature	0.001	1	0.001	0.94	0.366	Not significant
Residual	0.006	7	0.001			
Lack of fit	0.004	4	0.001	1.58	0.368	Not significant
Pure error	0.002	3	0.001			
Cor total	0.031	11				

Increasing the inlet air capacity decreased the yield as a result of complete drying (higher product temperature with higher inlet air capacity) and a consequent reduction in loss of power to the walls of the fluid-bed rather than the pellets.

### Box–Behnken design to obtain the response surface for particle size and percent yield

The BBD space and experiment results are shown in Table 4. A total of 17 runs were conducted to optimize the three parameters. The best-fit model was the quadratic model for each response. The coefficients of the quadratic models and the corresponding *p*-values are shown in Table 5.

**Table 4:** BBD with particle size and percent yield of coating of GLs-NS

Sample number	Experimental conditions			Results	
	Inlet air temperature (°C)	Inlet air capacity (Nm^3^)	Atomizing air pressure (bar)	*Y*_1_: particle size (nm)	*Y*_2_: percent yield
1	50	10	0.4	270.70	75.00
2	40	20	0.6	280.37	76.37
3	50	20	0.4	287.90	71.86
4	50	20	0.8	374.90	70.59
5	60	15	0.4	349.60	67.70
6	50	15	0.6	271.40	76.65
7	50	15	0.6	259.70	77.58
8	40	10	0.6	256.60	83.08
9	50	15	0.6	282.93	77.20
10	50	15	0.6	288.47	74.30
11	50	10	0.8	271.60	75.95
12	50	15	0.6	292.10	75.48
13	60	15	0.8	488.27	70.29
14	40	15	0.8	234.00	80.73
15	60	20	0.6	498.07	62.59
16	60	10	0.6	299.70	73.25
17	40	15	0.4	252.50	79.51

**Table 5:** Coefficients of the quadratic models and their corresponding *p*-values^a^

Source	*Y*_1_: particle size		*Y*_2_: percent yield		
	*F* value	*p*-value prob > *F*	*F* value	*p*-value prob > *F*	
Model	22.62	0.0002	15.07	0.0009	Significant
*A* – inlet air temperature	103.98	**<0.0001**	94.86	**<0.0001**	
*B* – intet air capacity	32.57	**0.0007**	30.19	**0.0009**	
*C* – atomizing air pressure	12.01	**0.0105**	0.55	0.4808	
*AB*	16.92	**0.0045**	1.41	0.2739	
*AC*	13.71	**0.0076**	0.17	0.6938	
*BC*	4.11	0.0821	0.44	0.5263	
*A* ^2^	16.71	**0.0046**	0.56	0.4794	
*B* ^2^	1.45	0.2670	4.99	0.0607	
*C* ^2^	0.91	0.3712	1.77	0.2253	
Lack of fit	4.61	0.0869	2.24	0.2255	Not significant

a Values in bold face represented significant terms (*p* < 0.05). *Y*_1_: correlation coefficient (*R*^2^) = 0.9668, adj. *R*^2^ = 0.9240. *Y*_2_: correlation coefficient (*R*^2^) = 0.9509, adj. *R*^2^ = 0.8878.

For the *Y*_1_ response, the experimental results were fitted into a second-order 300 polynomial equation:

3 Particle size = 278.92 + 76.52*A* + 42.83*B* + 26.01*C* + 43.65*AB* + 39.29*AC* + 21.52*BC* + 42.29*A*^2^ + 12.47*B*^2^ + 9.88*C*^2^

**Fig. 3:**
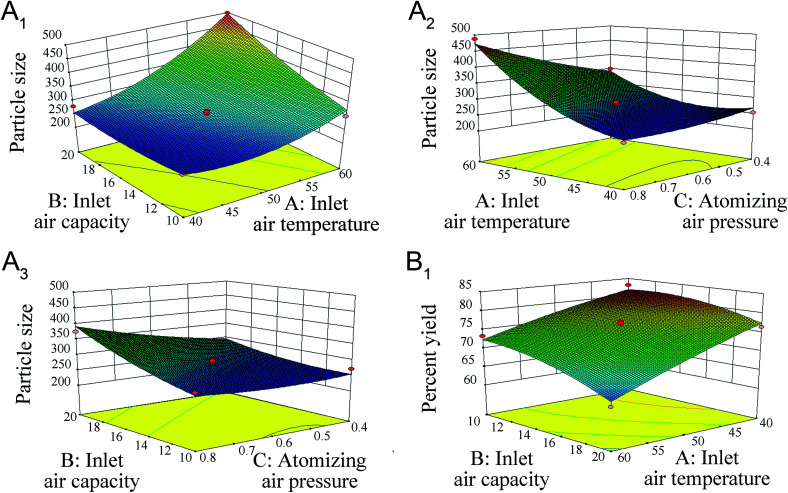
Response surface plots showing the effect of inlet air temperature (A), inlet air capacity (B) and atomizing air pressure (C) on particle size (A_1_–A_3_) and percent yield (B_1_) after fluid-bed coating of GLs-NS.

The regression coefficient values of eqn (3) are listed in Table 5. The inlet air temperature (*A*), inlet air capacity (*B*), atomizing air pressure (*C*), inlet air temperature × inlet air capacity (*AB*), inlet air temperature × atomizing air pressure (*AC*) and inlet air temperature × inlet air temperature (*A*^2^) parameters were significant at both *p* < 0.05 and *p* < 0.01. Three-dimensional response surface plots of the response variables of *Y*_1_ at different inlet air temperatures, inlet air capacities, and atomizing air pressures are shown in Fig. 3A_1_–A_3_, respectively.

For the *Y*_2_ response, the experimental results were fitted into a second-order polynomial equation:

4 Percent yield = 0.76–0.057A − 0.032B + 4.381E − 003C − 9.880E − 003AB + 3.415E − 003AC − 5.548E − 003BC − 6.059E − 003A^2^ − 0.018B^2^ − 0.011C^2^

In this case, A and B were significant model terms. The surface response for percent yield is shown in Fig. 3B_1_.

The values of the coefficient determination (*Y*_1_: *R*^2^ = 0.9668; *Y*_2_: *R*^2^ = 0.9509) and the adjusted coefficient determination (*Y*_1_: adj. *R*^2^ = 0.9240; *Y*_2_: adj. *R*^2^ = 0.8878) of the predicted model in this response indicates a high degree of correlation between the observed and predicted values. The usability of the model was assessed using the following optimal conditions: an inlet air temperature of 40 °C, an inlet air capacity of 11.6 Nm^3^ and an atomizing air pressure of 0.7 bar. Under the optimal conditions, the particle size and the percent yield of layered pellets were 254.3 ± 9.8 nm and 82.36 ± 1.87% (*n* = 3), respectively, consistent with the predicted values (236.34 nm and 81.86%) obtained from the model. Besides, the PDI, zeta-potential and drug content of the layered pellets also measured with 0.155 ± 0.02, −32.9 ± 3.8 mV and 10.88 ± 0.03% (*n* = 3). These results demonstrated the validity of the model. Then, the optimal conditions were selected to prepare 3 batches of products dried GLs-Ns.

### Characterization and *in vitro* evaluation of dried GLs-NS or layered pellets under optimum conditions

#### Redispersibility

Reconstitution is expressed by the redispersibility index (RDI). The mean particle size, PDI and zeta-potential of the liquid nanosuspensions prior to fluid-bed coating were 225.1 ± 7.3 nm, 0.165 ± 0.03, −34.9 ± 2.8 mV (*n* = 3), and 254.3 ± 9.8 nm, 0.155 ± 0.02, −32.9 ± 3.8 mV (*n* = 3) for fluid-bed coated GLs-NS, respectively. The RDI value of the particle size was 113 ± 4.4% (*n* = 3), indicating that the dried nanosuspensions possessed good redispersibility.

#### Solid state properties and morphology of the dried GLs-NS or layered pellets

Drug dissolution, absorption/bioavailability, and stability are greatly influenced by the form of the drug particle in the solid state. Herein, we assessed the solid-state form of the drug particles under the optimal conditions using PXRD and DSC.

**Fig. 4:**
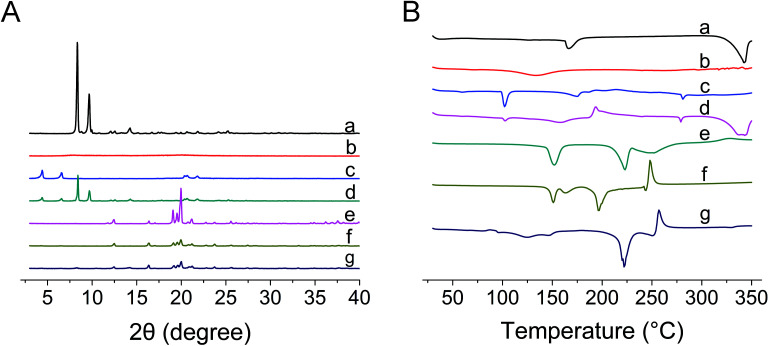
PXRD (A) and DSC (B) results of the raw GLs (a), HPMC (b), SDS (c), physical mixture of GLs with lactose (d), lactose (e), blank MCC pellet powder (f), layered pellet powder of GLs-NS (g).

As shown in Fig. 4A, the raw GLs showed diffraction peaks at 8.34 and 9.66, ranging from 2–40° (2*θ*), suggesting that the drug was highly crystalline in nature. The same diffraction peaks were also observed in samples of the physical mixture. However, the PXRD patterns for the dried GLs-NS prepared *via* fluid-bed coating did not show any diffraction peaks at the same temperature, suggesting that the drug particles were present in an amorphous state. The DSC pattern (Fig. 4B) confirmed the results obtained by PXRD. At approximately 343 °C, the endothermic peaks of the raw GLs and the physical mixture were observed, whereas the melting peak was absent from the dried nanosuspensions. However, another raw GLs melting peak (166 °C) disappeared from the thermograms of physical mixture, indicating that the melting peak of raw GLs could be influenced by the formulations. Based on the results of DSC and PXRD, the dried drug particles in the nanosuspensions prepared *via* fluid-bed coating are present in an amorphous state.

**Fig. 5:**
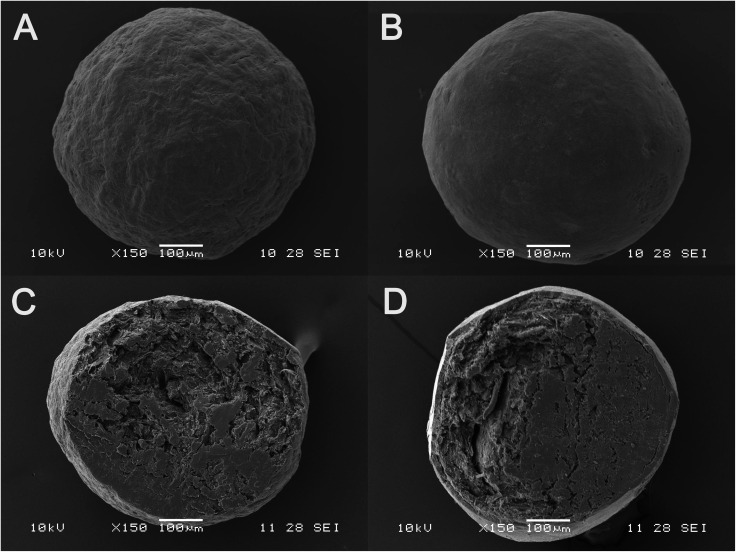
Sem micrographs of MCC pellets (A), layered pellets (B), cross-section of MCC pellets (C) and layered pellets (D).

The morphology of MCC pellets, the layered pellets and cross-section of MCC pellets and layered pellets under the optimal conditions using SEM is shown in Fig. 5. Compared with the MCC pellet (Fig. 5A), the surface of layered pellet (Fig. 5B) is smoother, indicating that the MCC pellet is tightly coated by GLs-NS. The cross-section of MCC pellet and layered pellet could be clearly observed in Fig. 5C and D. The cross-section view indicates a shell of compact coating of GLs-NS around the MCC pellets core (Fig. 5D).

#### *In vitro* dissolution

**Fig. 6:**
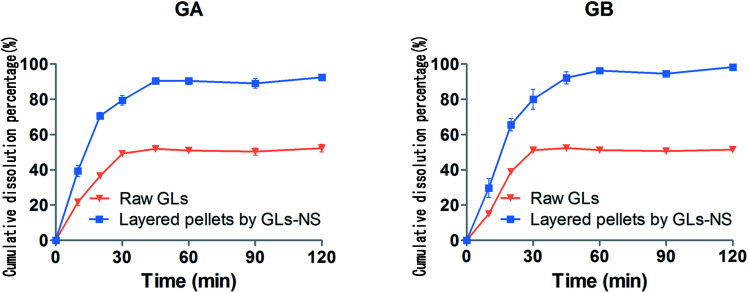
*In vitro* dissolution profiles for GA and GB from the raw GLs and layered pellets by GLs-NS.

For oral administration, the rapid dissolution originating from the increased specific surface area of drug nanosuspensions is generally regarded as a major advantage.^6^ The dissolution profiles of GA and GB form the raw GLs and layered pellets under the optimal conditions are shown in Fig. 6. Compared with the dissolution of the raw GLs, the layered pellets showed much faster dissolution during the first 30 min. In addition, the *in vitro* dissolution study demonstrated that both GA and GB could dissolve much more completely from the layered pellets than that from the raw drug powder (∼95% *vs.* ∼50% of drug release at the end point of the *in vitro* dissolution study). The increased dissolution observed in the layered pellets likely reflects two factors: (1) the mean particle sizes were reduced to the nanoscale range, which correspondingly increased the surface area available for dissolution (according to the Noyes–Whitney equation);^25^ (2) amorphous states were obtained, as indicated by the DSC and PXRD analyses.

## Conclusion

In the present study, dried GLs-NS was prepared by directly layering liquid nanosuspension onto the surface of MCC pellets using a fluid-bed process. A QbD approach was successfully applied to the fluid-bed coating of GLs-NS to obtain a better understanding of the fluid-bed coating process of nanosuspensions. The results showed that all three selected variables were significant factors (*p* < 0.05) affecting particle size. Higher inlet temperature, inlet air capacity or atomizing air pressure increased the particle size. Notably, the percent yield primarily depended on the inlet air temperature and inlet air capacity (*p* < 0.05). Higher percent yield was obtained with higher inlet air temperature or inlet air capacity. The optimal conditions for BBD, including inlet air temperature, inlet air capacity and atomizing air pressure, were set at 40 °C, 11.6 Nm^3^ and 0.7 bar, respectively. Compared with raw GLs, the products prepared under these optimum conditions were present in an amorphous state and possessed much faster dissolution. The particle size, percent yield, PDI, zeta-potential and redispersibility index of the product were determined as 254.3 ± 9.8 nm, 82.36 ± 1.87%, 0.155 ± 0.02, −32.9 ± 3.8 mV and 113 ± 4.4% (*n* = 3), respectively. These results suggested that fluid-bed coating technology following a QbD approach was suitable for the solidification of nanosuspensions.

## Conflicts of interest

The authors declare no conflicts of interest.

## Supplementary Material

## References

[cit1] Kesisoglou F., Panmai S., Wu Y. (2007). Adv. Drug Delivery Rev..

[cit2] Wang Y., Zhang L., Wang Q. W., Zhang D. R. (2013). J. Controlled Release.

[cit3] Wang B. H., Zhang W. B., Zhang W. (2005). Drying Technol..

[cit4] Müller R. H., Möschwitzer J., Bushrab F. N. (2006). Drugs Pharm. Sci..

[cit5] Lee J., Yu C. (2006). J. Controlled Release.

[cit6] Eerdenbrugh B. V., Mooter G. V. D., Augustijns P. (2008). Int. J. Pharm..

[cit7] He W., Lu Y., Qi J., Yin L., Wu W. (2013). Int. J. Nanomed..

[cit8] Dixit R., Puthli S. (2009). J. Pharm. Sci..

[cit9] Yao Q., Tao X. G., Tian B., Tang Y. L., Shao Y. J., Kou L. F. (2014). et al.. Colloids Surf., B.

[cit10] Luo Y., Xu L., Tao X., Xu M., Feng J., Tang X. (2013). Drug Dev. Ind. Pharm..

[cit11] Pieterjan K., Michaël A., Guy V. D. M. (2011). J. Pharm. Pharmacol..

[cit12] ICH , International Conference on Harmonisation (ICH) of technical requirements for registration of pharmaceuticals for human use, Topic Q9: Pharmaceutical quality system, Geneva, 2005

[cit13] ICH , International Conference on Harmonisation (ICH) of technical requirements for registration of pharmaceuticals for human use, Topic Q10: Pharmaceutical quality system, Geneva, 2008

[cit14] ICH , International Conference on Harmonisation (ICH) of technical requirements for registration of pharmaceuticals for human use, Topic Q8 (R2): Pharmaceutical development, Geneva, 2009

[cit15] Yu L. X. (2008). Pharm. Res..

[cit16] Tol T., Kadam N., Raotole N., Desai A., Samanta G. (2015). J. Chromatogr. A..

[cit17] Mcdermott M., Chatterjee S., Hu X., Ash-Shakoor A., Avery R., Belyaeva A. (2015). et al.. AAPS PharmSciTech.

[cit18] Beek T. A. V., Montoro P. (2009). J. Chromatogr. A..

[cit19] Maclennan K. M., Darlington C. L., Smith P. F. (2002). Prog. Neurobiol..

[cit20] Rui T. Q., Zhang L., Qiao H. Z., Huang P., Qian S., Li J. S. (2015). et al.. J. Pharm. Sci..

[cit21] Figueroa C. E., Bose S. (2013). Eur. J. Pharm. Biopharm..

[cit22] Parmentier J., Tan E. H., Low A., Möschwitzer J. P. (2017). Int. J. Pharm..

[cit23] Yue P. F., Wan J., Wang Y., Li Y., Ma Y. Q., Yang M. (2013). et al.. Int. J. Pharm..

[cit24] Chinese Pharmacopoeia Commission , Chinese Pharmacopoeia, Beijing China Medical Science Press, 2015

[cit25] Hattori Y., Haruna Y., Otsuka M. (2013). Colloids Surf., B.

